# Surgical Treatment for Localized Prostate Cancer: A Narrative Review of Literature

**DOI:** 10.3390/jcm14228144

**Published:** 2025-11-17

**Authors:** Vincenzo Salamone, Luca Mazzola, Francesco Lupo Conte, Francesca Conte, Beatrice Giustozzi, Marco Saladino, Daniele Paganelli, Giulia Carli, Filippo Lipparini, Gianni Vittori, Rino Oriti, Matteo Salvi, Luca Lambertini, Fabrizio Di Maida, Andrea Mari, Andrea Minervini, Antonio Andrea Grosso

**Affiliations:** Unit of Oncologic Minimally-Invasive Urology and Andrology, Department of Experimental and Clinical Medicine, University of Florence, Careggi Hospital, 50141 Florence, Italy; vincenzo.salamone@unifi.it (V.S.); luca.mazzola@unifi.it (L.M.); francescolupo.conte@unifi.it (F.L.C.); francesca.conte@unifi.it (F.C.); beatrice.giustozzi@unifi.it (B.G.); marco.saladino@unifi.it (M.S.); daniele.paganelli@unifi.it (D.P.); giulia.carli96@gmail.com (G.C.); filippo.lipparini@unifi.it (F.L.); giannivittori@yahoo.it (G.V.); rinooriti@yahoo.it (R.O.); m.salvi85@alice.it (M.S.); luca.lambertini@unifi.it (L.L.); fabridima90@gmail.com (F.D.M.); andreamari08@gmail.com (A.M.); andreamine@libero.it (A.M.)

**Keywords:** prostate cancer, robotic surgery, localized disease, prostatectomy, focal therapy

## Abstract

**Introduction**: Surgical management of prostate cancer has evolved significantly over more than a century, transitioning from open procedures to modern robot-assisted techniques. This narrative review traces the historical progression of surgical treatments for localized prostate cancer, from early approaches to the most recent advancements. **Materials and Methods**: An extensive literature search was conducted from 1 April to 1 June 2025, using PubMed and cross-referencing citations. The search gathered studies on the evolution of prostate cancer surgery, technical aspects, and comparisons between surgical and non-surgical treatments. Keywords included “PROSTATE CANCER”, “PROSTATE CANCER SURGICAL TREATMENT”, “RADICAL PROSTATECTOMY EVOLUTION”, “ROBOT-ASSISTED RADICAL PROSTATECTOMY”, and “FOCAL THERAPY AND PROSTATE CANCER”. **Results**: A total of 65 manuscripts, including original articles, systematic reviews, meta-analyses, and clinical trials focusing on the surgical treatment of localized prostate cancer, were selected. The review begins with the history of prostatic surgery, chronicling its evolution through laparoscopic and, ultimately, robotic approaches. It highlights how improved visualization and new technologies have led to better functional outcomes and preservation of structures while maintaining oncological safety. A specific focus is placed on the technical evolution of robot-assisted radical prostatectomy, new robotic platforms, and the role of focal therapy as an ultra-minimally invasive technique for localized disease. **Conclusions**: Robot-assisted radical prostatectomy represents the current reference surgical technique for localized prostate cancer. However, it is crucial to acknowledge RALP’s elevated cost and the need for further long-term, randomized data to establish definitive oncological equivalence compared to non-surgical options.

## 1. Introduction

Prostate cancer (PCa) is the second most commonly diagnosed cancer in men globally, with an estimated 1.4 million new cases in 2020 [1,2]. It is also the third leading cause of cancer-related death in men, with approximately 375,000 deaths reported in 2020. The incidence of PCa varies significantly by geographical region. The highest age-standardized rates (ASRs) per 100,000 are found in Australia/New Zealand (111.6) and Northern America (97.2), followed by Western and Northern Europe (94.9 and 85, respectively) [3,4]. Eastern and South–Central Asia report lower ASRs of 10.5 and 4.5, although these rates are increasing [4]. Age is a well-established, non-modifiable risk factor for PCa, with prevalence rising to 59% in men over 79 years old. However, clinical decision-making for screening, diagnosis, and treatment considers factors beyond age, including life expectancy, health status, frailty, and comorbidities. A life expectancy of ten years is a commonly used threshold for determining the benefit of local treatment [5].

Among the various local treatment options, radical prostatectomy (RP) remains a cornerstone for the surgical management of organ-confined PCa. Furthermore, RP has become a viable component of multi-modal therapy for locally advanced and oligometastatic PCa [6,7].

This paper provides a narrative review on the evolution of surgical treatment for organ-confined PCa, from the open era to modern robotic surgery. We explore the technical nuances of different approaches and their application in complex scenarios, such as locally advanced and oligometastatic disease. The review also examines key studies that compare surgical versus non-surgical primary treatments for PCa.

## 2. Materials and Methods

An extensive literature search was conducted utilizing the MEDLINE (via PubMed), Cochrane Central Register of Controlled Trials, and Web of Science (WoS) databases and citation cross-referencing. The search aimed to identify studies on the evolution of surgical treatment for prostate cancer, its technical aspects, and comparisons with non-surgical alternatives. Various combinations of the following terms were used: “PROSTATE CANCER”, “PROSTATE CANCER SURGICAL TREATMENT”, “ORGAN-CONFINED PROSTATE”, “RADICAL PROSTATECTOMY EVOLUTION”, “ROBOT-ASSISTED RADICAL PROSTATECTOMY”, “PROSTATE CANCER SURGICAL vs. NON-SURGICAL TREATMENT”, and “FOCAL THERAPY AND PROSTATE CANCER”. No time limit was considered in the literature search. Exclusion Criteria were: (a) Animal studies; (b) non-English articles; (c) Studies not meeting the quality criteria. Studies were subjected to a dual-review process. Each potentially eligible study underwent an initial assessment based on predefined quality criteria. These criteria included study design, sample size, statistical methods, and the clarity of reported outcomes. Studies failing to meet these criteria were excluded at this stage. The review process involved two independent reviewers (VS & LM) who conducted the initial screening and quality assessment. Any discrepancies or disagreements between reviewers regarding the eligibility or quality of a particular study were resolved through a third reviewer (FC). The synthesis and analysis summarized the main findings, identified patterns or trends across studies, and discussed the limitations and potential biases inherent in the included studies.

## 3. Results

**Figure 1:**
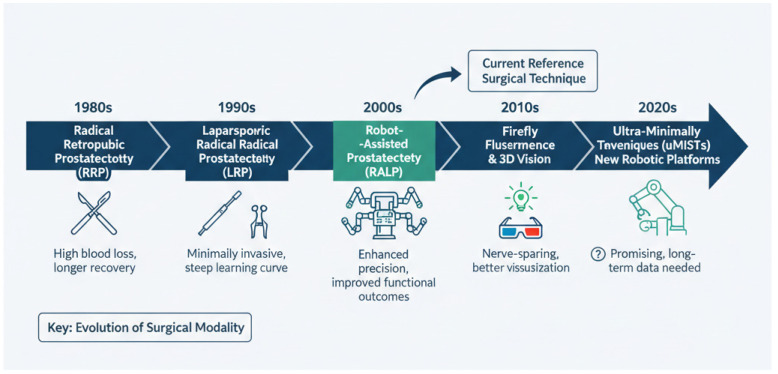
Timeline of evolution of radical prostatectomy approach.

Active treatment strategies for organ-confined prostate cancer (PCa) encompass both surgical and non-surgical modalities. The surgical management of this condition has undergone a significant evolution over the past century. Historically, the first partial prostatectomy was performed in 1867 by Billroth. Subsequently, in 1904, Young performed the first RP. The early 1990s marked the development of laparoscopic RP, which was followed in 2000 by Binder and Kramer’s first robot-assisted laparoscopic radical prostatectomy (RALP) [8,9] (Figure 1).

Non-surgical management options for organ-confined PCa include active surveillance (AS)/watchful waiting, radiotherapy (RT) with or without androgen deprivation therapy (ADT), and brachytherapy. In addition to these established methods, other minimally invasive modalities have emerged as potential treatment opportunities. These techniques, such as high-intensity focused ultrasound (HIFU) and transperineal laser ablation (TPLA), offer comparable oncological safety while aiming to reduce toxicity and improve functional outcomes. This review will specifically focus on these newer modalities.

The selection of a treatment strategy is guided by a comprehensive evaluation of various patient- and disease-specific factors. Preoperative counseling must integrate considerations of life expectancy, general health status, frailty, and disease stage and risk, along with the patient’s personal preferences, to determine the most appropriate course of treatment.

### 3.1. Surgical Treatment

#### Radical Prostatectomy

RP is a surgical procedure which consists of a demolitive and a reconstructive phase: the first step involves removing the entire prostate with its capsule and the seminal vescicles (SVs); further, the reconstructive phase is characterized by the vesico-urethral anastomosis which aims to restore the continuity between the urinary bladder and the urethra.

### 3.2. Open Radical Prostatectomy

Historically, a long and rich tradition of research has shaped the field of prostate surgery, as the surgical approach itself is a critical determinant of outcomes, particularly concerning complication rates. Between the 19th and early 20th centuries, nine distinct surgical approaches to the prostate were developed: suprapubic, retropubic, transpubic, infrapubic, transurethral, perineal, transrectal, ischiorectal, and sacral [10]. While many of these were quickly abandoned due to their invasiveness and limitations, the suprapubic, retropubic, and perineal approaches gained prominence. The first suprapubic prostatectomy operations were performed by Belfield and McGill, though these were partial resections [11]. The credit for the first successful suprapubic radical prostatectomy goes to Eugene Fuller, who performed the procedure in 1895 and documented it in his paper, “Six successful and successive cases of prostatectomy” [12].

In 1886, a German surgeon named Kuchler performed the first perineal radical prostatectomy. This technique was later refined by Hugh Young in 1904, whose published work in 1905 established it as the standard of care for over four decades [13]. A significant shift occurred in 1945 when Terence Millin introduced the retropubic prostatectomy for benign prostatic disease, publishing his initial report of 20 cases in *The Lancet* [14]. Millin aimed to address the high mortality associated with suprapubic procedures and the complications of the perineal approach, such as blood loss, incontinence, and urethro-rectal fistulae. He later applied these principles to radical prostatectomy for cancer, publishing his technique in 1947. However, due to significant blood loss and morbidity, only 7% of patients with localized prostate cancer underwent surgical treatment by the late 1970s [15]. The modern era of radical prostatectomy was ushered in by Patrick Walsh and José María Gil-Vernet, whose anatomical research on the dorsal venous complex and pelvic nerves provided a means to control surgical bleeding [16,17]. Walsh’s work focused on improving functional outcomes, including sexual function and urinary continence, without compromising oncological safety. His profound understanding of the neurovascular bundle’s relationship to the prostate led to the first nerve-sparing radical prostatectomy in 1982. This technique was subsequently described in a publication by Walsh, Lepor, and Egglestone, and his contributions remain the foundation of the modern retropubic radical prostatectomy (RRP) technique [18].

### 3.3. Laparoscopic Radical Prostatectomy

Historically, prostate surgery has also significantly evolved in its approach to the patient. The transition began with open radical prostatectomy and its subsequent refinements, leading to the establishment of laparoscopic radical prostatectomy (LRP) in the 1990s. The goal of LRP was to achieve outcomes comparable to open retropubic radical prostatectomy while reducing surgical morbidity [19,20]. After a report on its feasibility by Schuessler in 1997 and the standardization of the technique by Guillonneau et al. in 1999, LRP gained increasing interest within the urological community [19,20]. Like its open predecessor, LRP underwent several technical modifications, including the development of extraperitoneal versus transperitoneal and descending versus ascending approaches [21,22,23]. The initial intraperitoneal approach involved placing five trocars in a fan-like array, beginning with an umbilical trocar. This allowed for the transperitoneal development of the space of Retzius, providing access to the seminal vesicles and vas deferens. A later modification, the extraperitoneal approach, used a balloon dilator through the umbilical incision to create a pre-peritoneal space, allowing trocars to be placed without traversing the peritoneal cavity [24]. The theoretical advantages of this extraperitoneal technique include reduced operative time, a lower risk of postoperative ileus, and the prevention of abdominal organ interference or urine spillage into the abdominal cavity [25,26]. Regarding the surgical sequence, LRP was initially performed using a descending, posterior approach to dissect the seminal vesicles and vas deferens through the vesicorectal pouch. Today, most surgeons prefer an anterior approach, first transecting the bladder neck to access and dissect the seminal vesicles. This enhanced visualization allowed for better preservation of structures, which positively impacted both functional and oncologic outcomes compared to open retropubic radical prostatectomy.

### 3.4. Robot-Assisted Radical Prostatectomy

Robotic-assisted laparoscopic prostatectomy (RALP), first reported in 2000, has emerged as a significant advancement in the surgical management of prostate cancer [26,27,28]. Compared to traditional open and laparoscopic techniques, RALP offers improved ergonomics, a shorter learning curve, and enhanced visualization through 3D stereoscopic imaging and intuitive, tremor-filtered movements. This technological evolution represents a paradigm shift in urological surgery, leading to a new frontier for prostate cancer treatment. In the early 2000s, pioneering studies began to outline the techniques and initial outcomes of RALP. In 2002, Tewari et al. published a detailed description of their da Vinci robot-assisted anatomic radical prostatectomy [29]. This technique, built upon the principles of Walsh’s anatomic radical prostatectomy and the Montsouris laparoscopic approach, included a standardized patient and port setup. The surgical procedure was broken down into eleven distinct steps, from lymph node dissection to urethrovesical anastomosis, demonstrating the safety and reproducibility of the method for localized prostate cancer. The Vattikuti technique, later standardized by Shrivastava et al. [30] and Menon et al. [31], further refined the procedure, leading to excellent oncological and functional outcomes. Subsequent technical innovations, such as the fourth robotic arm and the extraperitoneal approach, have further enhanced results and reduced complications [32]. Extensive evidence has since accumulated from large series and long-term follow-up studies. A 2007 systematic review by Ficarra et al. established RALP as a feasible procedure associated with minimal blood loss, low complication rates, and shorter hospital stays [33]. The review also noted that positive surgical margin rates decreased with increased surgical experience, approaching those of retropubic and laparoscopic series. Subsequent papers, including systematic reviews and meta-analyses, provided more comprehensive outcome data [34,35,36,37]. These analyses highlighted significant advantages of RALP, including improved 12-month potency rates and faster urinary continence recovery compared to both RRP and LRP. Furthermore, they confirmed that RALP was associated with significantly lower blood loss and transfusion rates. While positive surgical margin rates were found to be comparable across all three techniques, data on cancer-specific survival and biochemical recurrence were considered preliminary at the time.

More recent analyses, such as the reverse systematic review by Moretti et al. involving over 1.3 million patients, have further substantiated these findings [38]. This review confirmed that minimally invasive surgery, and RALP in particular, yields superior perioperative outcomes compared to open surgery. With the exception of operative time, RALP demonstrated better results than both RRP and LRP in terms of estimated blood loss, transfusion rates, hospital stay, catheterization time, and overall complication rates.

Collectively, the improved visualization and precise dissection techniques afforded by robotic surgery have not only enhanced anatomical understanding but have also led to superior functional outcomes and comparable oncological outcomes when compared to RRP and LRP. While the superior perioperative and functional outcomes of RALP are well-established, the primary goal of radical prostatectomy remains definitive cancer eradication. Evaluating RALP’s oncologic efficacy requires a rigorous examination of positive surgical margin (PSM) rates, biochemical recurrence (BCR), and long-term cancer-specific survival (CSS). PSMs, defined as the presence of tumor cells at the inked surface of the resected specimen, are a crucial short-term oncologic endpoint associated with an increased risk of BCR and the need for secondary treatments [39]. In 2012, Richard Gaston proposed a lateral approach to maximize bladder neck preservation when performing RARP [40]. For all these considerations, RALP has become the predominant approach for localized prostate cancer.

### 3.5. Retzius-Sparing vs. Antegrade RALP

Building on the foundational Vattikuti and extraperitoneal techniques, the robotic era has seen the development of diverse surgical approaches to prostatectomy. One such innovation is the Retzius-sparing radical prostatectomy (RSRP). First presented by Galfano et al. in 2013, this technique involves accessing the prostate through the pouch of Douglas, thereby avoiding the structures in the Retzius space critical for continence and potency [41].

The initial series of 200 patients treated with RSRP demonstrated significant functional benefits [42]. The results showed a notably high rate of early urinary continence, with over 90% of patients continent within seven days of catheter removal. The study also reported promising early potency outcomes, as 40% of patients who underwent a full intrafascial nerve-sparing procedure were able to engage in sexual intercourse within one month post-surgery. However, a major limitation of this initial series was a high overall positive surgical margin (PSM) rate of 25.5%, with rates of 22% for pT2 disease and 45.2% for pT3 disease, with most margins located at the prostatic apex.

Subsequent systematic reviews have confirmed the functional advantages of RSRP. A 2020 comparative analysis by Checcucci et al. affirmed that RSRP is a safe and feasible alternative to standard RALP, offering faster and superior recovery of continence in both the short and long term without increasing complication risks [43]. While concerns about higher PSM rates persisted, studies have shown that these rates decrease as surgeons progress through their learning curve. For instance, Galfano et al. later demonstrated a statistically significant reduction in PSM rates after the first 100 procedures [41].

Efforts have been made to address the issue of apical PSMs, a common concern in RSRP. The “collar technique,” described by Bianchi et al., involves a distal incision of the urethral sphincter complex to reduce apical PSMs [44]. This method led to a significant decrease in PSMs to 0% in pT2 cases. A multicenter study by Galfano et al. on high-risk patients further corroborated the functional benefits, reporting an 84% continence rate at one year. However, oncological challenges remained, with an overall PSM rate of 22.1% and a biochemical recurrence rate of 27.5% at a median follow-up of 22 months [45].

### 3.6. RALP and New Robotic Platforms

In an effort to mitigate the high costs associated with traditional robotic systems, several novel multiport robotic platforms have emerged. A systematic review by Reitano et al. encompassing 74 studies and 5467 patients investigated the perioperative, functional, and oncological outcomes of nine such platforms [46]. The review, which included 41 studies on RALP, found that the Hugo RAS was the most frequently studied new system. When comparing these new platforms to the established Da Vinci system, oncological outcomes appeared similar. A pooled analysis from 14 comparative studies showed no significant heterogeneity in positive surgical margin (PSM) rates or biochemical recurrence (BCR) at three months. Similarly, functional outcomes, particularly urinary continence at three months, were comparable between the new platforms and the Da Vinci system. Limited data on potency rates for the new platforms, primarily from two studies on the Hugo RAS, showed comparable outcomes to the Da Vinci system. While system failures and errors were initially reported with some of the new platforms, these issues were generally resolved through software updates and surgeon learning curves [47,48].

In addition to new multiport systems, the advent of the Da Vinci single-port (SP) platform has further refined RALP [49]. This system allows for complex procedures through a single incision, potentially reducing invasiveness and improving recovery, particularly for frail patients or those with hostile abdomens. The SP system’s articulated instruments and enhanced triangulation enable a supine, extraperitoneal approach, which may enhance surgical recovery without compromising oncological safety [50,51,52,53].

A meta-analysis by Xiao et al. compared SP-RALP with multiport (MP)-RALP [54]. The findings indicated that SP-RALP was associated with several key advantages, including reduced estimated blood loss, shorter hospital stays, and earlier catheter removal, suggesting improved hemostatic control and enhanced postoperative recovery. Moreover, patients undergoing SP-RALP required significantly fewer opioids, highlighting a benefit in postoperative pain management. Importantly, the analysis found no significant differences between the two techniques in terms of operative time, nerve-sparing rates, or major complications. Both PSM and BCR rates were comparable, supporting the equivalent oncological safety of SP-RALP. While further high-quality, long-term studies are needed, SP-RALP represents a promising, safe, and effective alternative for prostate cancer treatment.

### 3.7. The Role of Lymph Node Dissection During a RALP

Pelvic lymph node dissection (PLND) is the standard for surgical nodal staging in prostate cancer (PCa), providing crucial information on the extent of disease and guiding postoperative management [55,56]. Although its therapeutic benefit remains debated in randomized trials and SRs [57,58], PLND is associated with an increased risk of complications, longer operative times, and extended hospital stays [59]. Therefore, it is typically reserved for patients with a high risk of lymph node involvement. Historically, the selection of PLND candidates relied on preoperative nomograms (e.g., MSKCC, Briganti) that use clinical features to estimate the risk of lymph node metastasis (LNM). However, these tools were developed before the widespread adoption of next-generation imaging, which has higher sensitivity. Prostate-specific membrane antigen (PSMA) positron emission tomography (PET), for instance, has demonstrated superior accuracy in detecting nodal and distant metastases compared to conventional imaging and boasts a negative predictive value of up to 98% in low-risk men [60].

The introduction of PSMA-PET has prompted the development of new risk stratification tools, such as the updated Briganti and Amsterdam–Brisbane–Sydney nomograms, which integrate this advanced imaging to enhance predictive accuracy [61,62]. Given the high negative predictive value of PSMA-PET, recent research has explored whether PLND can be safely omitted in patients with negative preoperative PSMA-PET (miN0). This is based on the premise that traditional risk calculators, developed in the conventional imaging era, might overestimate the risk of LNM in these patients. In a recent study, Gandaglia et al. externally validated and compared existing tools to predict LNM in miN0 PCa patients [63]. Their findings demonstrated that the Briganti 2023 [63] nomogram outperformed other models, accurately predicting LNM and identifying a substantial number of patients who could safely avoid an unnecessary PLND. This work highlights how integrating molecular imaging into risk assessment can optimize PLND recommendations and improve the clinical management of PCa.

### 3.8. Non-Surgical Treatment

Besides RP and RT, other modalities have emerged as potential therapeutic treatments for localized PCa, aiming for combining equivalent oncologic outcomes with minimal invasiveness and, therefore, reducing toxicity and potential complications and improving functional outcomes.

### 3.9. Focal Therapy

The detection of prostate cancer (PCa) at earlier stages has spurred the development of focal therapy (FT), a strategy that aims to balance cancer control with the preservation of functional outcomes.

A 2025 systematic review by Slusarczyk et al. provided a comprehensive analysis of FT for localized PCa, investigating seven different energy sources [64]. The review, which included 50 studies, predominantly featured high-intensity focused ultrasound (HIFU) and laser ablation. The findings demonstrated that FT provides effective short-term oncological control, favorable intermediate-term radical treatment-free survival, and a commendable safety profile. Importantly, FT was associated with superior preservation of urinary continence and erectile function compared to radical prostatectomy (RP), although long-term oncological data are still pending.

A key study supporting these findings is the prospective, noninferiority HIFI trial by Ploussard et al. [65]. This trial compared whole-gland or subtotal HIFU with RP in over 3300 patients from 46 centers. The primary endpoint, salvage therapy-free survival (STFS) at 30 months, was 90% in the HIFU group versus 86% in the RP group. Noninferiority analysis indicated that HIFU was associated with a lower risk of needing salvage therapy. Furthermore, HIFU demonstrated significantly less impairment of urinary continence and erectile function, as measured by the International Index of Erectile Function (IIEF-5), highlighting its functional benefits.

Transperineal laser ablation (TPLA) is another ultra-minimally invasive surgical technique (uMIST) gaining attention for PCa treatment [66,67]. A 2025 systematic review by Polverino et al. on TPLA for localized PCa included ten studies, demonstrating its use across a range of risk categories [68,69]. While a post-treatment drop in PSA was consistently observed, the rate of residual cancer varied widely, from 4% to 57%, with clinically significant cancer detected in up to 31% of cases. The need for secondary treatment ranged from 7% to 30%. The overall complication rate was variable (0% to 66%) but generally involved mild, transient events. A notable advantage was the consistent preservation of functional outcomes, both in the short and long term.

Overall, while early results for FT are promising, particularly regarding functional preservation, further research is needed to standardize patient selection criteria and establish long-term oncological efficacy through randomized trials comparing uMIST with standard-of-care treatments.

## 4. Limitations of the Review

This narrative review, while providing a comprehensive overview of the evolution and current status of surgical treatment in localized PCa, inherently carries certain limitations. As a non-systematic review, it does not employ the exhaustive search strategy and rigorous selection criteria characteristic of a systematic review or meta-analysis, which may introduce reporting bias regarding the studies selected for discussion. Furthermore, a significant portion of the evidence base regarding novel surgical techniques and comparative outcomes, particularly for functional endpoints and emerging focal therapies, originates from non-randomized studies, observational cohorts, or single-institution series. These study designs are susceptible to selection bias, confounding by indication, and varying levels of surgeon experience, which can influence reported results and limit the generalizability of findings, particularly when inferring definitive equivalence or superiority across different treatment modalities.

### Practice Implication

The evidence reviewed herein offers several important implications for contemporary urological practice and multidisciplinary team discussions. For patients considering radical prostatectomy, urologists can confidently counsel on the superior perioperative and functional recovery expectations associated with minimally invasive approaches, particularly regarding early continence and potency preservation compared to traditional open surgery. Crucially, for patients exploring less invasive options, focal therapies like TPLA should be discussed primarily within the context of ongoing clinical trials or institutional registries, emphasizing the need for long-term oncological and functional data before widespread adoption outside of these frameworks. This ensures that patients receive care aligned with current evidence while contributing to the robust evaluation of emerging technologies.

## 5. Conclusions

Surgical management of localized prostate cancer has undergone a century of evolution, transitioning from open partial prostatectomies to RP, with each phase leading to new anatomical discoveries and improved outcomes. The late 20th century saw the introduction of LRP, which demonstrated the benefits of minimally invasive surgery, providing comparable oncological results with improved perioperative and functional outcomes compared to open RP.

The dawn of the 21st century marked a significant paradigm shift with the advent of RALP. This technology enhanced surgical precision through improved visualization and dexterity, leading to a more profound understanding of prostatic anatomy. This heightened knowledge, in turn, facilitated superior functional outcomes—particularly in preserving continence and potency—while maintaining oncological control on par with open and laparoscopic approaches. Consequently, RALP, by demonstrating superior functional and perioperative outcomes compared to open and laparoscopic methods, has become the dominant surgical approach for localized prostate cancer. Major consensus guidelines present both RALP and external beam radiotherapy as standard primary treatments for localized disease. While the functional and short-term oncological results for RALP are excellent, its clinical application must be viewed with caveats, including the high cost of robotic systems, the steep learning curve for surgeons, and the continued need for mature, long-term oncologic equivalence data when compared to both radical non-surgical treatments and emerging uMISTs. Preliminary results for uMISTs are promising regarding functional preservation, yet long-term oncological efficacy requires confirmation through prospective, randomized trials comparing them with standard-of-care treatments.

## Data Availability

No new data were created for this manuscript.

## Abbreviations

The following abbreviations are used in this manuscript:

PCa	Prostate cancer
RP	Radical Prostatectomy
ADT	Androgen Deprivation Therapy
AS	Active Surveillance
RT	Radiotherapy
HIFU	High-intensity Focused Ultrasound
TPLA	Transperineal Laser Ablation
SV	Seminal Vesicle
RRP	retropubic radical prostatectomy
LRP	laparoscopic radical prostatectomy
RALP	Robotic-assisted laparoscopic prostatectomy
RSRP	Retzius-sparing radical prostatectomy
PSM	positive surgical margin
BCR	biochemical recurrence
SP	single-port
PLND	Pelvic lymph node dissection
LNM	lymph node metastasis
PSMA	Prostate-specific membrane antigen
PET	positron emission tomography
FT	Focal therapy
uMIST	ultra-minimally invasive surgical technique
